# Minimally invasive tubular resection of spinal arachnoid webs

**DOI:** 10.1016/j.bas.2026.106079

**Published:** 2026-05-02

**Authors:** Florian Volz, Jürgen Beck, Ulrich Hubbe, Amir El Rahal, Christoph Scholz, Marc Hohenhaus, Ralf Watzlawick, Marco Bissolo, Lucas Becker, Marius Schwabenland, Jan-Helge Klingler

**Affiliations:** a Department of Neurosurgery, Medical Center University of Freiburg, Freiburg im Breisgau, Germany; b Faculty of Medicine, University of Geneva, Geneva, Switzerland; c Department of Neuroradiology, Medical Center University of Freiburg, Freiburg im Breisgau, Germany; d Institute of Neuropathology, Faculty of Medicine, University of Freiburg, Freiburg im Breisgau, Germany

**Keywords:** Spinal arachnoid web, Minimally invasive surgery, Tubular approach

## Abstract

**Introduction:**

Spinal arachnoid webs (SAWs) are rare intradural extramedullary lesions causing progressive myelopathy. For surgical resection, minimally invasive approaches, especially using tubular retractors, remain underreported.

**Research question:**

This study presents a series of minimally invasive SAW resections performed through expandable and non-expandable tubular retractors.

**Material and methods:**

This retrospective monocentric cohort study included patients with minimally invasive tubular resection of SAWs between July 2017 and December 2025. Preoperative and postoperative status was assessed using the modified McCormick grading scale, surgical details were descriptively analyzed.

**Results:**

The cohort comprised 5 male and 7 female patients (mean age 61.1 ± 8.8 years). All patients presented with progressive myelopathy. All lesions were located in the upper thoracic spine, with T2-3 being most common. All patients demonstrated the pathognomonic scalpel sign on preoperative MRI, two patients (17%) had associated syringomyelia. Surgical technique involved expandable (3/12) and non-expandable tubular retractors (20 mm diameter, 9/12). Mean operative time was 110 ± 30 min with minimal blood loss in all cases. Two patients required revision surgery. No permanent neurological deficits occurred, and all patients maintained stable or improved neurological function postoperatively.

**Discussion and conclusion:**

This series represents the largest experience with tubular resection of SAWs using non-expandable retractor systems with 20 mm diameter access corridors. The revision rate aligns with the published literature and reflects the technical challenges rather than a limitation of the approach. This truly minimally invasive approach is safe and effective in appropriately selected patients and in experienced centers.

## Highlights

• Twelve consecutive patients with surgical treatment of spinal arachnoid webs (SAW).
• First study of minimally invasive approaches with non-expandable tubular retractors.
• This truly minimally invasive approach is technically feasible and safe.
• Outcomes are comparable to traditional open techniques.

## Abbreviations:

BMI: Body Mass Index
CSF: Cerebrospinal fluid
EMA: Epithelial membrane antigen
MRI: Magnetic resonance imaging
SAW: Spinal arachnoid web

## 1 Introduction

Spinal arachnoid webs (SAWs) represent a distinct form of focal arachnopathy characterized by intradural fibrous bands that typically arise from the posterior or posterolateral arachnoid membrane. These lesions most commonly occur in the thoracic spine and can lead to progressive myelopathy through direct compression of the spinal cord or alteration of cerebrospinal fluid (CSF) dynamics (Nisson et al., 2019; Voglis et al., 2022; Na et al., 2025). Although still considered rare, increasing awareness and improved imaging techniques have led to more frequent recognition of this entity.

The pathognomonic radiological feature of SAWs is the “scalpel sign” on sagittal T2-weighted MRI, which represents focal dorsal indentation of the spinal cord corresponding to the site of arachnoid web attachment (Reardon et al., 2013). This imaging finding, combined with characteristic clinical presentation of progressive thoracic myelopathy, typically establishes the diagnosis. Associated syringomyelia may be present in a subset of patients (Na et al., 2025; Adib et al., 2023).

Surgical resection remains the definitive treatment for symptomatic SAWs, with the goal of decompressing the spinal cord and restoring normal CSF flow dynamics. Traditional open (hemi-)laminectomy or laminoplasty approaches have demonstrated excellent outcomes with high rates of neurological improvement (Nisson et al., 2019; Voglis et al., 2022; Na et al., 2025). However, open approaches involve extensive muscle dissection, removal of posterior elements, and potential for iatrogenic instability or chronic pain.

The emergence of minimally invasive spinal surgery techniques has revolutionized the approach to intradural pathology. Tubular retractor systems, initially developed for discectomy procedures (Clark et al., 2017), have been successfully adapted for intradural extramedullary lesions. Experience from our center with minimally invasive approaches to other intradural pathologies, including spinal hemangioblastomas (Krüger et al., 2019), spinal schwannomas and neurofibromas (Argiti et al., 2024), and cerebrospinal fluid leaks in spontaneous intracranial hypotension (Beck et al., 2022; Volz et al., 2024), has demonstrated the feasibility and advantages of these techniques. Our institutional experience also includes innovations such as double-tubular techniques (Hubbe et al., 2022). These systems offer the reported advantages of reduced muscle trauma, decreased blood loss, and preserved posterior spinal elements while maintaining adequate surgical access and visualization (Wong et al., 2015; Helal et al., 2022; Vergara and Barone, 2018; Dauleac et al., 2021).

However, data on minimally invasive approaches specifically for spinal arachnoid webs remain limited and no series has specifically reported outcomes using non-expandable tubular retractors for SAW resection, representing a truly minimally invasive approach. This study presents the first case series of minimally invasive tubular resection of SAWs, with emphasis on the technical feasibility and safety of non-expandable retractor systems in this challenging pathology.

## 2 Methods

### 2.1 Study design

This retrospective single-center study included all consecutive patients who underwent minimally invasive tubular resection of thoracic spinal arachnoid webs between July 2017 and December 2025. The study was conducted after approval from the local ethics committee (25-1325-S1-retro). Individual informed consent was waived due to the strictly retrospective design in accordance with institutional guidelines.

### 2.2 Preoperative assessment

All patients underwent comprehensive neurological examination and high-resolution MRI of the thoracic spine. The presence of the characteristic “scalpel sign” on sagittal T2-weighted images was documented in all cases. Preoperative neurological status was assessed using the modified McCormick grading scale (McCormick et al., 1990).

### 2.3 Surgical technique

**Fig. 1:**
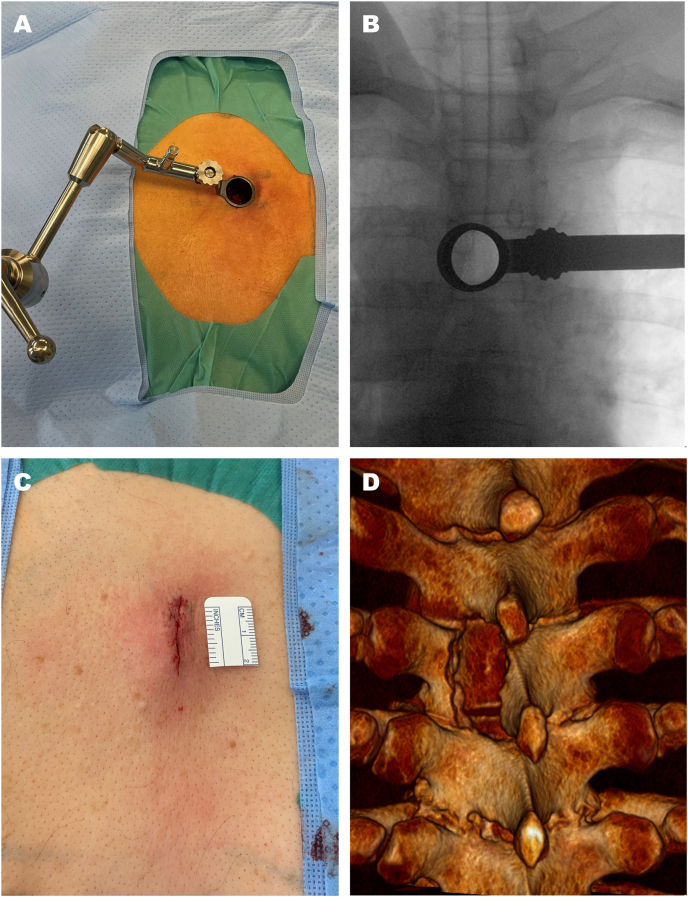
**(A)** Non-expandable 20-mm tubular retractor mounted to the operating table via an adjustable and maneuverable holding arm. **(B)** Slightly oblique intraoperative anteroposterior fluoroscopic view obtained through the non-expandable 20-mm tubular retractor, confirming correct positioning of the minimally invasive access system. Note the deliberately oblique fluoroscopy view straight through the tubular retractor to ensure that the intended segment is exactly in the tubular working trajectory. **(C)** Approximately 2.5 cm skin incision. **(D)** Postoperative three-dimensional reconstructed CT scan demonstrating the tailored bony approach, in this case via hemilaminectomy.

**Fig. 2:**
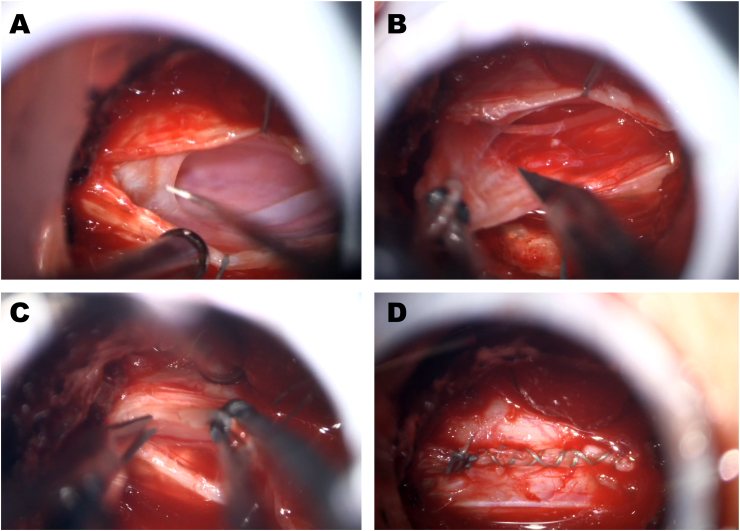
Intraoperative view through a 20-mm non-expandable tubular retractor. **(A)** The longitudinally opened dura is retracted laterally using sutures (green). Intradural dissection of the arachnoid web is performed using a microhook. **(B, C)** Resection of the arachnoid web using curved microscissors. **(D)** Watertight dural closure with suturing.

All procedures were performed under general anesthesia in prone position, either with rigid head fixation in the Mayfield-Clamp or on a dedicated horseshoe gel headrest. The surgical technique employed either non-expandable (METRx system, Medtronic, Fig. 1) or expandable (Quadrant system, Medtronic) tubular retractor systems. For non-expandable systems, after fluoroscopic level confirmation, a 2.5 cm median skin incision was made; for expandable systems, skin incision could have been extended up to 3-4 cm. After a unilateral, blunt transmuscular dilation with sequential dilators followed the insertion of the non-expandable (20 mm diameter with individually adjusted length) or expandable tubular retractor. A targeted hemilaminotomy was performed to expose the dura. The dura was opened longitudinally, and the arachnoid web was resected using bayonet microinstruments (Fig. 2, Video 1). After complete resection and confirmation of pulsatile CSF flow, the dura was closed in watertight fashion via suturing and extradural fibrin-patch augmentation. Intraoperative ultrasound was used at the discretion of the operating surgeon. Intraoperative neuromonitoring was not routinely used. Postoperatively no prolonged bed rest was prescribed and patients were permitted to begin mobilization 8 h after surgery.

### 2.4 Outcome measures

Primary outcome was change in neurological status using the modified McCormick scale at the last clinical follow up. Secondary outcomes included operative time, blood loss, complications, revision surgery, and length of stay after surgery. Results were reported descriptively stating the mean and standard deviation.

## 3 Results

### 3.1 Patient demographics

Twelve consecutive patients underwent surgery between July 2017 and December 2025 (Table 1). The cohort comprised 5 male and 7 female patients with mean age of 61.1 ± 8.8 years (range 50-80). Mean Body Mass Index was 29.3 ± 5.2 kg/m^2^ (range 21.1-36.2) All patients presented with progressive thoracic myelopathy with a mean symptom duration of 14.3 ± 12.9 months (range 4-51).

**Table 1:** Patient characteristics and surgical details.

No.	Sex	Age (years)	BMI (kg/m^2^)	Level	Retractor Type	Time (minutes)	Length of stay	McCormick preoperative	McCormick postoperative	Follow-up (days)	Revision
1	F	80	25.1	T4-5	expandable	151	11	I	I	1900	Yes
2	F	62	26.2	T2-3	expandable	141	5	II	No follow up	xx	No
3	M	50	29.1	T2-3	expandable	113	5	II	I	89	No
4	M	75	31.4	T6-7	non-expandable (20 × 40mm)	141	5	I	I	1164	No
5	M	58	36.2	T1-2	non-expandable (20 × 50mm)	117	6	I	I	773	Yes
6	F	54	35.8	T2-3	non-expandable (20 × 40mm)	101	4	III	I	243	No
7	F	58	21.9	T5-6	non-expandable (20 × 30mm)	83	4	II	I	204	No
8	M	62	26.6	T2-3	non-expandable (20 × 40mm)	142	4	II	I	110	No
9	F	51	21.1	T3-4	non-expandable (20 × 30mm)	81	4	I	I	104	No
10	M	59	33.8	T2-3	non-expandable (20 × 50mm)	111	4	III	I	91	No
11	F	61	30.9	T4-5	non-expandable (20 × 40mm)	66	4	II	II	60	No
12	F	63	33.9	T2-3	non-expandable (20 × 50mm)	73	3	II	I	45	No

### 3.2 Radiological findings

All lesions were thoracic (T1-T7), most common at T2-3 (6/12, 50%). The scalpel sign was present in all patients (100%), two patients (17%) had associated syringomyelia.

### 3.3 Surgical details and tubular retractor systems

**Fig. 3:**
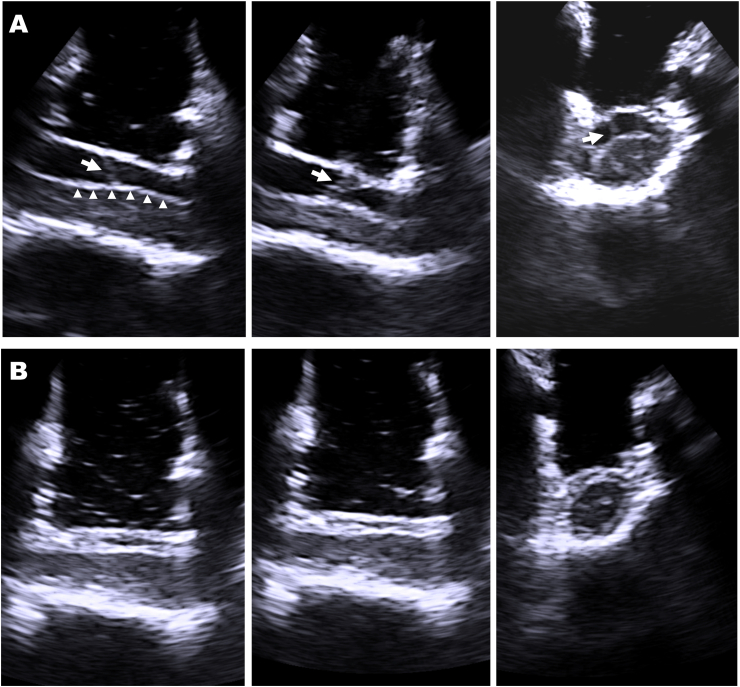
Intraoperative ultrasound using a 10 × 8.6-mm (burr-hole) transducer introduced through a non-expandable tubular retractor. **(A)** Before dural opening, the arrow indicates the posterior arachnoid web, which moves with cerebrospinal fluid (CSF) pulsation and obstructs free CSF passage. The triangles denote the posterior aspect of the spinal cord, which is pushed anteriorly by the arachnoid web, representing an intraoperative appearance of the characteristic “scalpel sign”. **(B)** After resection of the arachnoid web and closure of the dura, (1) CSF passage is re-established, (2) the spinal cord is fully re-expanded, and (3) the spinal cord is no longer fixed anteriorly representing a recession of the “scalpel sign”.

Mean operative time was 110 ± 30 min (range 66-151). In the first three patients in the study period (25%) expandable tubular retractors were used. For the subsequent nine patients (75%) non-expandable tubular retractors (all 20 mm diameter, length varying between 30 and 50 mm). Documented blood loss was 200 ml in one case and less than 100 ml in all other cases. Intraoperative ultrasound with a 10 × 8.6 mm (burr hole) transducer was applied in 5 cases with non-expandable tubular retractors. This provided a good intraoperative visualization and confirmation of the complete resection of the SAW, even through the 20 mm tube (Fig. 3).

### 3.4 Clinical outcomes and complications

**Fig. 4:**
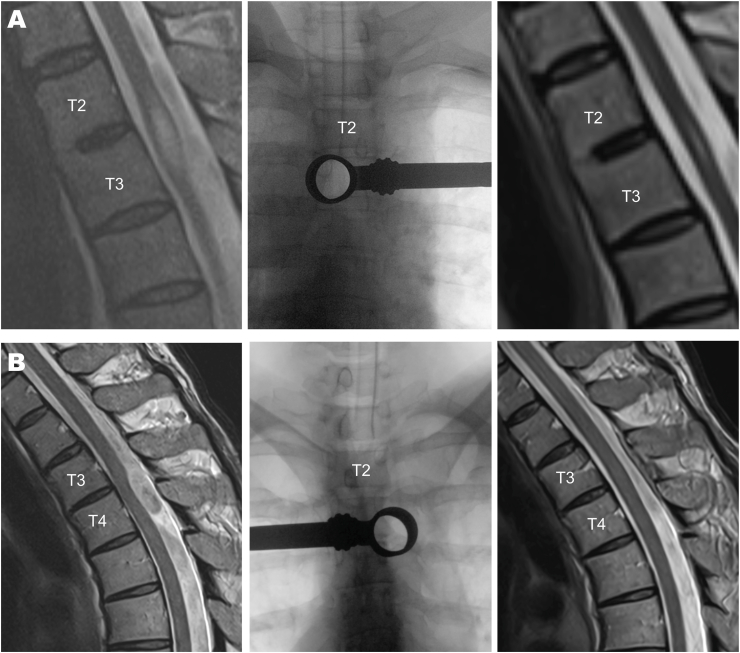
Pre- and postoperative MRI (left and right column) as well intraoperative fluoroscopy (middle column) in two illustrative cases. **(A)** Preoperative MRI shows the scalpel sign at T3 and a rather large syrinx. The non-expandable tubular retractor (diameter 20 mm, length 50 mm) is deliberately centered just below the left T3 pedicle. The slight oblique fluoroscopy view straight through the retractor ensures the correct working trajectory. Postoperative MRI shows the receding ventral displacement of the spinal cord and regression of the syrinx. **(B)** Preoperative MRI shows the scalpel sign at the disc space T3-4 without a syrinx. The tubular working trajectory is aimed at the disc space confirmed via deliberately angulated fluoroscopy. Postoperative MRI shows completely receding scalpel sign and no more ventral displacement of the spinal cord.

Mean length of stay after surgery was 4.9 ± 2.1 days (range 3-11). The one case (female, 80 years of age) with an extraordinary long postoperative stay of 11 days was prolonged by a pre-existing chronic pain syndrome and domestic care issues, unrelated to the surgical procedure. In all patients the final examination prior to discharge showed no neurological deterioration compared to the preoperative assessment. Follow-up time after surgery was at least 6 weeks with a wide range of up to 5 years. For one patient no clinical follow-up data was available. All patients demonstrated stable or improved neurological function. Two patients (17%) required revision surgery: One patient (male, 75 years) 50 days after the primary surgery because of a CSF fistula, which presented with small subdural hygromas. After closure of the postoperative leak the hygromas resolved without further treatment. One patient (female, 80 years) did not show up for the recommended control MRI after surgery. The MRI three years after the primary surgery demonstrated a persisting SAW, which in retrospect might rather be considered as an incomplete resection during the first procedure. Except for this one case, the routine MRI 6-12 weeks the after surgery showed successful resection of the SAW with regression of the ventral displacement of the spinal cord and regression of the syrinx, if present preoperatively (Fig. 4).

### 3.5 Histopathological findings

**Fig. 5:**
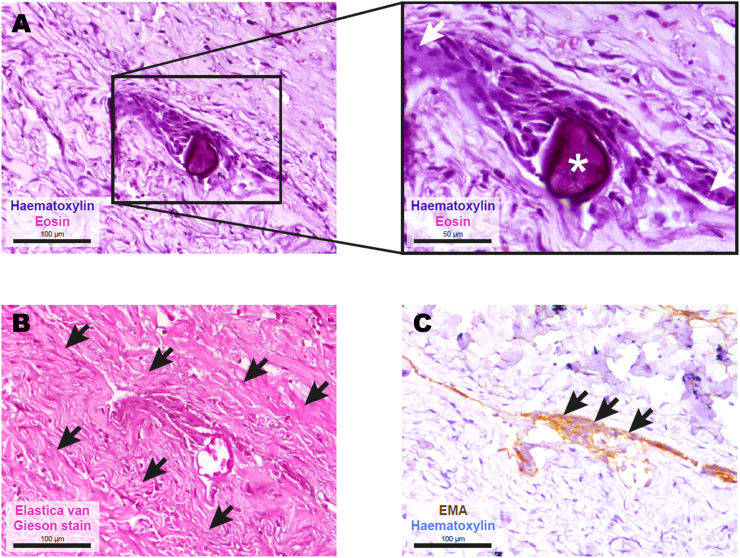
Histopathological findings in patients with spinal arachnoid web (SAW). **(A)** Representative hematoxylin and eosin (H&E)-stained section of formalin-fixed, paraffin-embedded (FFPE) tissue showing clusters of arachnoid cap cells with round to elongated, predominantly chromatin-dense nuclei. Occasional nuclear holes (arrows) and psammoma bodies (asterisks) are observed. Scale bar: 100 μm (left panel) and 50 μm in the magnification (right panel). **(B)** Elastica van Gieson staining reveals that these meningothelial cell clusters are embedded into collagen-rich connective tissue. Scale bar: 100 μm. **(C)** Immunohistochemical reaction for epithelial membrane antigen (EMA, brown) highlights the arachnoidal origin of the cells. Counterstaining with hematoxylin (light blue). Scale bar: 100 μm.

Typical findings included clusters of arachnoid cap cells with round to elongated, predominantly chromatin-dense nuclei (Fig. 5A). Some nuclei presented with nuclear holes, a histomorphological hallmark of arachnoid cells. Moreover, psammoma bodies were occasionally present and constitute another hallmark feature of arachnoidal tissue. As demonstrated by Elastica van Gieson staining, these small groups of arachnoid cells were typically embedded into a collagen-rich connective tissue matrix (Fig. 5B). Immunohistochemical staining for epithelial membrane antigen (EMA Fig. 5C) further corroborated the arachnoidal origin of these cell clusters (Schwabenland et al., 2021).

## 4 Discussion

This study represents the first reported case series of minimally invasive tubular resection of spinal arachnoid webs using predominantly non-expandable retractor systems with 20 mm diameter access corridors. Our experience demonstrates that this truly minimally invasive approach is technically feasible and achieves outcomes comparable to traditional techniques.

### 4.1 Significance of non-expandable tubular retractor systems

A distinct feature of our series is the predominant use of non-expandable tubular retractors (9/12 patients, 75%) with a fixed diameter 20 mm. Previous case reports by Vergara and Barrone (n = 2) and Dauleac et al. (n = 1) utilized expandable systems with larger corridors (Vergara and Barone, 2018; Dauleac et al., 2021). Our experience demonstrates that non-expandable retractors with maximum 20 mm diameter provide adequate access for safe and complete resection. In addition to the smaller skin incision, the completely blunt access trajectories of non-expandable tubular retractors offer the theoretical advantage of further reduced muscle trauma when no expansion of the retractor is applied. However, due to the limited case number, no systematic comparison between the two techniques was possible.

Our institutional expertise with minimally invasive approaches to other intradural pathologies includes experience with spinal hemangioblastomas (Krüger et al., 2019), schwannomas and neurofibromas (Argiti et al., 2024), as well as CSF leak repairs in spontaneous intracranial hypotension (Beck et al., 2022; Volz et al., 2023, 2024). This facilitated the successful application of the tubular technique to spinal arachnoid webs (SAW). As sufficient experience is mandatory, a general recommendation for minimally invasive approaches is definitely not advisable and traditional approaches (e.g. with speculum-type retractors) are a viable alternative.

The tubular approach presented here, regardless of expandable or non-expandable, is a unilateral and blunt technique. It avoids extensive muscle dissection and removal of stabilizing posterior elements (Fig. 1D) but still offers a sufficient working trajectory. In contrast, in the series by Voglis et al., 10 out of 12 patients were operated via a mono- or multi-segmental laminectomy (Voglis et al., 2022). The advantages of an unilateral approach, which preserves the midline structures and muscles, are well known from various pathologies and biomechanical studies (Wong et al., 2015; Helal et al., 2022; Healy et al., 2014; Oda et al., 2002).

A frequently cited argument against the use of minimally invasive tubular approaches is the alleged inability to securely close the dura by suturing. In accordance with the studies cited above on various intradural pathologies (Krüger et al., 2019; Argiti et al., 2024; Beck et al., 2022; Volz et al., 2023, 2024), and with a study on accidental durotomies in minimally invasive tubular lumbar fusion (Klingler et al., 2015), the current series once again demonstrated that watertight dural suturing can be achieved with tubular approaches (Fig. 2D). When using appropriately small ultrasound transducers, intraoperative ultrasound can also be applied to confirm the surgical goal (in this case, resection of the SAW and restoration of cerebrospinal fluid passage, but also resection of intramedullary tumors (Krüger et al., 2019)), further arguing for small tubular approaches (Fig. 3).

### 4.2 Revision rate

Due to the limited number of cases, no statistical analysis of risk factors for a potentially required revision was performed. The reported 17% revision rate (two out of twelve cases) aligns to that reported in several comparable series of arachnoid cysts, with revision rates between 14 and 20% (Krings et al., 2001; Moses et al., 2018; Mohindra et al., 2010). The need for a revision surgery therefor might be considered more as a characteristic feature of the technically challenging SAW surgery rather than a limitation of the minimally invasive approach. Notably, no revisions were necessary in the last four years of the study period, which could be interpreted as increasing surgical experience in terms of an internal learning curve. We believe, that with continuously growing experience and systematic use of intraoperative imaging techniques such as ultrasound and endoscopic assistance, the revision rate can be reduced even further. As in other rare conditions, this trend advocates for a centralized treatment in appropriately experienced centers.

### 4.3 Limitations

The retrospective single-center design experience, and the small sample size limit generalizability. A comparison between non-expandable (n = 9) and expandable (n = 3) systems is limited by small numbers.

## 5 Conclusion

This study presents the first case series of minimally invasive tubular resection of SAWs using predominantly non-expandable retractor systems with maximum 20 mm diameter. Our series demonstrates that fixed-diameter non-expandable systems can provide adequate access while maximizing tissue preservation. In experienced centers, minimally invasive tubular resection of SAWs using non-expandable retractors represents a safe and effective alternative to traditional open surgery.

## Availability of data and material

On request.

## Ethical approval

25-1325-S1-retro.

## Author's contributions

Study design: FV JHK; Data acquisition: FV, JHK, LB, MS; MB; Infrastructure: JB, UH, FV, JHK, MS; Data analysis: FV, JHK; Graphical illustrations: FV; Videos: AER; Manuscript writing: FV JHK with contributions of all authors. Manuscript corrections: contributions of all authors.

## Funding or financial support

No author or institution received financial support for this article's research, authorship, or publication.

## Declaration of competing interest

The authors declare that they have no known competing financial interests or personal relationships that could have appeared to influence the work reported in this paper.
